# Birth Weight, Ethnicity, and Exposure to Trihalomethanes and Haloacetic Acids in Drinking Water during Pregnancy in the Born in Bradford Cohort

**DOI:** 10.1289/ehp.1409480

**Published:** 2015-09-04

**Authors:** Rachel B. Smith, Susan C. Edwards, Nicky Best, John Wright, Mark J. Nieuwenhuijsen, Mireille B. Toledano

**Affiliations:** 1 MRC-PHE (Medical Research Council–Public Health England) Centre for Environment and Health, Department of Epidemiology and Biostatistics, School of Public Health, Imperial College London, St Mary’s Campus, Norfolk Place, London, United Kingdom; 2 Bradford Institute for Health Research, Bradford Royal Infirmary, Bradford, United Kingdom; 3 Center for Research in Environmental Epidemiology (CREAL), IMIM (Hospital del Mar Medical Research Institute), CIBERESP (Centro de Investigación Biomédica en Red de Epidemiología y Salud Pública), Barcelona, Spain

## Abstract

**Background:**

Evidence for a relationship between trihalomethane (THM) or haloacetic acid (HAA) exposure and adverse fetal growth is inconsistent. Disinfection by-products exist as complex mixtures in water supplies, but THMs and HAAs have typically been examined separately.

**Objectives:**

We investigated joint exposure at the individual level to THMs and HAAs in relation to birth weight in the multi-ethnic Born in Bradford birth cohort.

**Methods:**

Pregnant women reported their water consumption and activities via questionnaire. These data were combined with area-level THM and HAA concentrations to estimate integrated uptake of THMs into blood and HAA ingestion, accounting for boiling/filtering. We examined the relationship between THM and HAA exposures and birth weight of up to 7,438 singleton term babies using multiple linear regression, stratified by ethnicity.

**Results:**

Among Pakistani-origin infants, mean birth weight was significantly lower in association with the highest versus lowest tertiles of integrated THM uptake (e.g., –53.7 g; 95% CI: –89.9, –17.5 for ≥ 1.82 vs. < 1.05 μg/day of total THM) and there were significant trends (p < 0.01) across increasing tertiles, but there were no associations among white British infants. Neither ingestion of HAAs alone or jointly with THMs was associated with birth weight. Estimated THM uptake via showering, bathing, and swimming was significantly associated with lower birth weight in Pakistani-origin infants, when adjusting for THM and HAA ingestion via water consumption.

**Conclusions:**

To our knowledge, this is the largest DBP and fetal growth study to date with individual water use data, and the first to examine individual-level estimates of joint THM–HAA exposure. Our findings demonstrate associations between THM, but not HAA, exposure during pregnancy and reduced birth weight, but suggest this differs by ethnicity. This study suggests that THMs are not acting as a proxy for HAAs, or vice-versa.

**Citation:**

Smith RB, Edwards SC, Best N, Wright J, Nieuwenhuijsen MJ, Toledano MB. 2016. Birth weight, ethnicity, and exposure to trihalomethanes and haloacetic acids in drinking water during pregnancy in the Born in Bradford cohort. Environ Health Perspect 124:681–689; http://dx.doi.org/10.1289/ehp.1409480

## Introduction

During drinking-water treatment, disinfectants such as chlorine react with natural organic matter and bromide ions present in the water, producing disinfection by-products (DBPs) (IPCS 2000). There are > 600 different species of DBPs including trihalomethanes (THMs), haloacetic acids (HAAs), haloacetonitriles, halofuranones, and nitrosamines (Richardson et al. 2007). THMs [comprising chloroform, bromodichloromethane (BDCM), dibromochloromethane (DBCM), and bromoform] are the most prevalent class in drinking waters, followed by HAAs (comprising bromo-, chloro-, dibromo-, dichloro-, tribromo-, trichloro-, bromochloro-, bromodichloro-, and dibromochloro-acetic acids) (Krasner et al. 1989). THMs are regulated at 100 μg/L per sample in the United Kingdom (DWI 2010), whereas HAAs are currently unregulated.

Exposure to volatile THMs via showering and bathing plays an influential role in determining total blood dose via dermal exposure and inhalation, whereas ingestion plays a relatively minor role (Backer et al. 2000; Nuckols et al. 2005). Inhalation and dermal routes are expected to contribute little to uptake of nonvolatile HAAs (Xu et al. 2002; Xu and Weisel 2003), making ingestion the dominant route for HAA exposure.

The relationship between DBPs and fetal growth outcomes has been investigated in > 30 epidemiological studies since the early 1990s, but the evidence for an association with THMs and also with HAAs is inconsistent. However, some studies suggest that brominated species may be most relevant (Costet et al. 2012; Rivera-Núñez and Wright 2013).

Epidemiological studies have typically examined THMs and HAAs in isolation, but these are not necessarily good proxies for each other, nor for other DBP classes. In reality, DBPs exist as complex mixtures in drinking-water supplies, which may be geographically specific, thus contributing to inconsistent findings in this research field. One recent epidemiological study has examined THM–HAA mixtures using area-level exposure data (Rivera-Núñez and Wright 2013); however, to our knowledge, there are no studies to date incorporating individual water use into DBP exposure assessment that have addressed THM–HAA mixture effects.

In this study we aimed to investigate, for the first time, potential effects of joint THM–HAA exposures, incorporating individual estimates of uptake, on birth weight and ethnic differences in the Born in Bradford (BiB) prospective birth cohort.

## Methods

*Study population.* Born in Bradford (BiB) is a longitudinal multi-ethnic birth cohort study aiming to examine the impact of environmental, psychological, and genetic factors on maternal and child health and well-being (Wright et al. 2013). Bradford is a city in the north of England with high levels of socioeconomic deprivation and ethnic diversity. Approximately half of the births in the city are to mothers of South Asian origin. Women were recruited while waiting for their glucose tolerance test, a routine procedure offered to all pregnant women registered at the Bradford Royal Infirmary, at 26–28 weeks gestation. The only exclusion criterion was if a woman planned to move away from Bradford before the birth (Raynor and Born in Bradford Collaborative Group 2008). For those providing informed consent to participate in BiB, a baseline questionnaire was completed via an interview with a study administrator. The baseline questionnaire was transliterated into Urdu and Mirpuri, and questionnaires in Mirpuri were administered by trained bilingual interviewers because it has no written form (Raynor and Born in Bradford Collaborative Group 2008). Information on birth weight, gestational age, and health during pregnancy was obtained from clinical records.

The full BiB cohort recruited 12,453 women during 13,776 pregnancies between 2007 and 2010. The BiB cohort represents 64% of all pregnancies at Bradford Royal Infirmary and is broadly characteristic of the city’s maternal population (Wright et al. 2013). Ethical approval for the data collection was granted by Bradford Research Ethics Committee (Ref 07/H1302/112).

Our initial data set contained 13,525 babies for whom clinical record data were available. We restricted the data set to 13,199 singleton births. For mothers with repeated pregnancies in BiB, we randomly selected one of these pregnancies (excluding 1,271 babies). We excluded 2,100 due to missing water-use data, 98 for whom THM exposure could not be calculated for all trimesters, 1 record with missing birth weight, and 531 preterm births. This left 9,198 babies eligible for the analysis. After taking into account missing covariate data, final models included up to 7,438 babies.

*Exposure assessment.* Water use. The baseline questionnaire ascertained typical daily consumption of tap water, bottled water, tea, coffee, and squash (concentrated diluting juice, including any other drinks made up with tap water) at home, work/study, or elsewhere; water filtering at home and work; and frequency and duration of showering, bathing, and swimming. We calculated daily consumption (liters) of cold tap water (sum of tap water and squash), hot beverages made from tap water (sum of tea and coffee), and total tap water (sum of tap water, squash, tea, coffee) and minutes spent showering, bathing, and swimming per week.

THM and HAA concentrations in drinking water. Routine monitoring data on THMs and other parameters were provided by Yorkshire Water, for the eight water supply zones (WSZ) covering the study area from January 2006 to March 2011. Each WSZ was sampled nine times per year on average, giving 374 data points in total. Additional samples for HAA analysis were taken quarterly from these eight WSZs, by Yorkshire Water staff alongside their routine sampling regime, between June 2007 and November 2010. The additional samples were picked up by study staff, and analyzed for HAAs. HAAs were analyzed using a modified form of U.S. Environmental Protection Agency (EPA) Method 552.3 (Tung et al. 2006; U.S. EPA 2003). The derivatized HAAs (methyl esters) were measured using gas chromatography with microelectron capture detection (Agilent 6890; Santa Clara, CA, USA).

Predictive modeling of both THMs and HAAs was undertaken to obtain estimates of DBP concentrations for times and places for which data were sparse, in line with previous studies (Nieuwenhuijsen et al. 2008; Toledano et al. 2005). Log-transformed THMs were modeled using linear regression, with a spline in month, a factor for year, and a factor for WSZ in order to provide monthly WSZ-specific concentrations. THM samples below the limit of detection (LOD) were assigned a value equal to half the LOD, per Malliarou et al. (2005). Bromoform was not modeled individually because so many data points were below the LOD; instead total brominated THMs (THMBr) were modeled.

Only three HAAs had sufficient detectable data points to be modeled: 158 points each for dichloroacetic acid (DCAA) and for trichloroacetic acid (TCAA) and 143 data points for bromodichloroacetic acid (BDCAA). Model selection for each HAA was first performed in a frequentist framework in R 2.15.2 (R Core Team 2012). Square root–transformed DCAA and TCAA and log-transformed BDCAA were modeled, with all models including a factor for WSZ and a spline on time. Models selected additionally included the following variables: conductivity (all HAA models), temperature (DCAA and TCAA models only), TOC (TCAA model only), and total chlorine (BDCAA model only). To account for parameter uncertainty and impute missing covariate data, the best model for each HAA was then run in a Bayesian framework in WinBUGS 1.4.3 (Lunn et al. 2000) to predict mean DCAA, TCAA, and BDCAA concentration levels by WSZ and year-quarter. All HAA data points for quarter 2 of 2009 were anomalously low compared with data for all other sampling quarters, suggesting possible unreliability of the laboratory analysis data for this quarter. Therefore, data from quarter 2 of 2009 were excluded, and the Bayesian model was allowed to predict HAA values for this quarter. In addition, the Bayesian model was allowed to predict HAA values for the first quarters of 2007 and 2011 (i.e., extrapolating to one additional quarter either side of the HAA sampling window), to cover as many women’s pregnancies as possible, but without overstretching the model.

Time-weighted average DBP concentrations. Each woman was assigned modeled THM and HAA concentrations (micrograms per liter) for the WSZ encompassing her residence postcode at time of recruitment, and additionally for her workplace postcode if applicable and possible, within a GIS (geographic information system) via WSZ-postcode link. Workplace exposure could not be assigned to employed women if workplace address/postcode data was missing or insufficient to allow geocoding (*n* = 1,157) or if a woman’s workplace was outside the WSZs included in the exposure assessment (*n* = 514). Time-weighted average concentrations were calculated for each woman. The first trimester was defined as days 1–93, the second trimester as days 94–186, and the third as day 187 to the day preceding delivery. The time weighting was based on the proportion of the whole pregnancy or trimester falling into each month (THMs) or quarter (HAAs). Each mother was thus assigned concentrations (micrograms per liter) for total trihalomethane (TTHM), chloroform, BDCM, DBCM, THMBr, DCAA, TCAA, and BDCAA for each trimester/whole pregnancy. TTHM, DCAA, TCAA, and BDCAA were summed to give DBP7 concentration, a joint THM–HAA exposure metric for use in regression models. For 15% of women, time-weighted average HAA concentrations could not be calculated for their pregnancy because the pregnancy commenced before January 2007 (the earliest extent of the HAA models). For those women assigned workplace concentrations (*n* = 2,353; 58.4% of 4,024 employed women), weightings of 40/112 and 72/112 were applied to work and residence, respectively, based on 8 hr at work 5 days per week, out of a total of 112 waking hours per week.

Integrated uptake of THMs in blood via multiple activities. Time-weighted average THM concentrations (micrograms per liter) were multiplied by individual water use and THM uptake factors [micrograms/(micrograms per liter)liter for consumption or micrograms/(micrograms per liter)min for showering/bathing] to estimate whole pregnancy and trimester-specific average THM uptake into blood (micrograms/day) for each activity (water consumption, showering, bathing). THM uptake factors were calculated from biomonitoring studies (Aggazzotti et al. 1995; Backer et al. 2000; Lynberg et al. 2001) or based on uptake factors previously used in the literature (Villanueva et al. 2007). Further details are given in Supplemental Material, “THM uptake factors,” and uptake factor values are shown in Supplemental Material, Table S1. Uptake calculations for swimming used swimming pool THM concentrations (Chu and Nieuwenhuijsen 2002). Uptakes for each activity were summed to give the exposure metric of integrated uptake—total blood dose, for chloroform, BDCM, DBCM, THMBr, and TTHM. We also calculated integrated TTHM uptake via showering, bathing, and swimming as a separate exposure metric so that this could be included in a model with DBP7 (sum of TTHM and HAA3) ingestion via water consumption.

Ingestion of THMs and HAAs via drinking water consumption. Time-weighted average TTHM and HAA concentrations (micrograms per liter) were multiplied by water consumption (liters/day) to calculate the exposure metrics of whole pregnancy and trimester-specific ingestion via water consumption (micrograms/day) of TTHM, DCAA, TCAA, BDCAA, HAA3 (sum of DCAA, TCAA, BDCAA), and DBP7, the latter being an individual-level metric of joint THM–HAA exposure.

For both integrated THM uptakes and THM–HAA ingestion metrics, boiling adjustment factors were applied to beverages made with hot water (THMs: –92%; DCAA +43.5%; TCAA –36.9%; BDCAA –56.5%) and filtering reduction factors applied to cold filtered tap water (THMs –90%; DCAA –61.8%; TCAA –67.4%; and BDCAA –78.5%) (Edwards 2014; Smith 2011). All other drinks (including squash) were treated as unfiltered.

*Statistical analysis.* We analyzed continuous birth weight because this had greatest statistical power compared with term low birth weight (LBW) and small for gestational age (SGA) and because the latter outcomes do not necessarily identify pathologically small infants comparably for white British and Pakistani-origin subpopulations because of different underlying birth weight distributions (Moser et al. 2008). Multiple linear regressions examining the relationship between continuous birth weight and categorical exposure metrics (using tertiles of THM or HAA exposure metrics and using individual water use activities were categorized as follows: cold tap water consumption: 0.0–0.4 L/day, 0.6–1.0 L/day, 1.2–1.4 L/day, ≥ 1.6L/day; showering: 0 min/week, 1–60 min/week, 61–105 min/week, > 105 min/week; bathing: 0 min/week, 1–60 min/week, 61–120 min/week, > 120 min/week; swimming: yes vs. no) were run in STATA 12.1 (StataCorp, College Station, TX, USA).

To examine joint THM–HAA exposure, we included two exposure terms [DBP7 ingestion via water consumption and integrated TTHM uptake via showering, bathing, and swimming (both as tertiles)] in the model. We chose to examine joint exposure in this way because, to our knowledge, there is no information available from which HAA uptake factors can be calculated, and thus integrated DBP7 uptake across all activities could not be estimated. To incorporate THM and HAA exposures on a comparable scale within models, a joint THM–HAA exposure term was estimated as DBP7 ingestion via water consumption (because ingestion is the dominant route for HAA exposure), with the remainder of exposure included as a separate term for TTHM uptake via showering/bathing/swimming (because HAA exposure via these activities is negligible).

We also analyzed the relationship between time-weighted area-level DBP7 exposure (as tertiles) and continuous birth weight, to investigate whether any relationship could be observed between birth weight and DBP levels in tap water, that is, without taking into account individual-level water consumption/activities. This model was included to aid interpretation of models examining individual-level DBP exposures, to help rule out potential confounding by unmeasured factors related to water consumption or water-related activities.

All models were adjusted *a priori* for 10 maternal factors [caffeine intake (≥ 200 mg/day vs. < 200 mg/day), education (highest educational qualification categorized as no qualifications, school, further education, higher education, other), fasting and post-load glucose from oral glucose tolerance test (continuous), ethnicity (categorized as white British, Pakistani origin, other), smoking (categorized as never smoker, ever smoker, current smoker), parity (categorized as 0, 1, ≥ 2), age (continuous), body mass index (BMI) at recruitment as quartiles (because prepregnancy BMI was not available), Index of Multiple Deprivation (IMD) 2010 quintiles of deprivation (McLennan et al. 2011)] and two infant factors (gestational age at delivery as linear and quadratic terms, sex).

Spearman’s correlations between exposure metrics were calculated.

Models were run for total population and subgroup analyses stratifying by the two largest ethnic groups white British and Pakistani-origin. The ethnic category “other” comprised a number of ethnicities and was not considered meaningful to examine because of its small size and lack of cultural cohesiveness.

We considered *p* < 0.05 statistically significant. The *F*-test was used to *a*) test whether the categorical exposure term, as a whole, was significant within regression models (giving a *p*-value for overall significance), and *b*) to test whether exposure–ethnicity interaction terms were, as a whole, significant within regression models (giving a *p*-value for interaction). *p*-Values for a linear trend across exposure categories were derived by including the exposure tertiles (coded as 0, 1, 2) as a continuous variable in the model.

Because HAA ingestion is driven by water consumption (which may change during pregnancy) and may be sensitive to filtering assumptions, we conducted a sensitivity analysis for trimester 2 only (because water consumption data were collected at 26–28 weeks, and may be more reflective of behavior during the second trimester), applying stricter filtering criteria to calculation of HAA ingestion. Sensitivity analyses additionally adjusting models for environmental tobacco smoke and language of questionnaire completion were conducted because these variables showed the greatest difference between Pakistani-origin women in the highest THM uptake tertile versus those in the lower tertiles. Overall pattern and direction of relationship, and magnitude of any change in point estimates, were examined to determine if conclusions were robust or sensitive to changes in the model.

## Results

White British (40%) and Pakistani-origin (44%) women are the two predominant ethnic groups in the population, and there are differences in birth weight outcomes, employment status, deprivation, parity, diabetes, smoking, and alcohol and caffeine consumption between the two groups (Table 1). Compared with white British women, women of Pakistani origin drink less water from all sources combined, spend less time bathing but more time showering, and very few go swimming (2% vs. 14% white British). There were few or no missing data on maternal age, employment, education, IMD quintile, or smoking, but approximately 3–4% of the population were missing data on parity, BMI, fasting and post-load glucose levels, and 8.9% were missing data on caffeine consumption, which reduced numbers in final regression models. Time-weighted average DBP concentrations in tap water to which mothers were exposed differed by ethnicity, but only marginally and not in any consistent direction (Table 2). However, estimates of THM uptakes and HAA ingestion are lower on average for Pakistani-origin women compared with white British women, reflecting differences in water use between the two groups. Individual-level exposure metrics within the THM class and within the HAA class were highly correlated (see Supplemental Material, Table S2).

**Table 1:** Birth outcomes and maternal characteristics.

Variable	Total (*n* = 9,198)			White British (*n* = 3,665, 40%)			Pakistani origin (*n* = 4,098, 45%)			Ethnicity *p*-value^*c*^
	%^*a*^ or mean ± SD	% missing data^*b*^	% undertaking activity	%^*a*^ or mean ± SD	% missing data^*b*^	% undertaking activity	%^*a*^ or mean ± SD	% missing data^*b*^	% undertaking activity	
Birth outcomes
Term birth weight (g)	3,295 ± 484	0		3,424 ± 482	0		3,186 ± 462	0		< 0.001
Term LBW^*d*^	4	0		2	0		6	0		< 0.001
SGA	12	0		8	0		16	0		< 0.001
Gestational age (weeks)	39.8 ± 1.2	0		40.0 ± 1.2	0		39.7 ± 1.2	0		< 0.001
Maternal characteristics
Maternal age (years)		0			0			0		< 0.001
< 25	33			28			36
25–29	32			38			28
30–34	23			20			24
≥ 35	13			13			13
Employed (yes)	44	0.1		64	0.03		23	0.1		< 0.001
Highest educational qualification		0.2			0.1			0.2		< 0.001
No qualifications	21			20			26
School	31			34			31
Further education	15			17			13
Higher education	25			19			26
Other	8			10			4
IMD quintile^*e*^		0.2			0.2			0.2		< 0.001
1 (most deprived)	66			50			79
2	18			22			14
3	11			18			6
4	3			6			0
5 (least deprived)	2			3			0
Parity		4.0			3.5			4.3		< 0.001
0	40			48			31
1	27			29			24
≥ 2	29			19			40
BMI at recruitment (kg/m^2^)	28.3 ± 5.5	3.6		28.9 ± 5.8	3.0		27.9 ± 5.2	4.1		< 0.001
Gestational diabetes	8	3.8		5	3.9		11	3.8		< 0.001
Fasting glucose (mmol/L)	4.5 ± 0.5	4.1		4.4 ± 0.4	4.3		4.6 ± 0.6	3.8		< 0.001
2 hr post-load glucose (mmol/L)	5.7 ± 1.5	4.1		5.4 ± 1.3	4.3		5.9 ± 1.6	3.8		< 0.001
Current smoker	14	0.04		28	0.1		3	0.05		< 0.001
ETS during pregnancy	32	0.4		43	0.3		25	0.5		< 0.001
Alcohol consumption^*f*^	16	3.1		34	6.8		0.2	0.1		< 0.001
Caffeine ≥ 200 mg/day^*g*^	17	8.9		31	7.4		6	9.6		< 0.001
Water use
Consumption (L/day)
Cold tap water	1.14 ± 0.81	0	94	1.11 ± 0.94	0	91	1.18 ± 0.65	0	98	< 0.001
Hot tap water	0.50 ± 0.58	0	81	0.72 ± 0.75	0	81	0.33 ± 0.31	0	81	< 0.001
Total tap water	1.64 ± 0.95	0	99	1.84 ± 1.14	0	99	1.51 ± 0.71	0	100	< 0.001
Bottled water	0.29 ± 0.54	0	35	0.40 ± 0.61	0	47	0.15 ± 0.38	0	21	< 0.001
All water	1.92 ± 1.03	0	100	2.23 ± 1.20	0	100	1.66 ± 0.77	0	100	< 0.001
Showering (min/week)^*h*^	93 ± 74	0	72	86 ± 64	0	71	94 ± 78	0	69	< 0.001
Bathing (min/week)^*h*^	123 ± 119	0	68	151 ± 142	0	76	96 ± 81	0	66	< 0.001
Combined showering/bathing (min/week)	151 ± 126	0	100	175 ± 139	0	100	128 ± 107	0	100	< 0.001
Swimming (min/week)^*h*^	73 ± 47	0	7	72 ± 49	0	4	75 ± 38	0	2	0.62
Abbreviations: ETS, environmental tobacco smoke; IMD, Index of Multiple Deprivation; LBW, low birth weight; SGA, small for gestational age. ^***a***^Percentage in category. ^***b***^Percentage missing out of total column *n*. ^***c***^*p*-Value for *t*-test and chi-square test for continuous and categorical variables respectively, comparing white British vs. Pakistani-origin. ^***d***^Birth weight < 2,500 g and gestation ≥ 37 weeks. ^***e***^Index of Multiple Deprivation (IMD) quintiles–quintiles of relative deprivation at Lower Super Output Area (LSOA) level across England. ^***f***^Three months before and during pregnancy. ^***g***^In 4 weeks preceding questionnaire. ^***h***^Mean, SD, and *p*-value calculated only for those undertaking the activity.										

**Table 2:** Maternal DBP exposures.

Variable	Total (*n* = 9,198)		White British (*n* = 3,665, 40%)		Pakistani origin (*n *= 4,098, 45%)		Ethnicity *p*-value^*b*^
	Mean ± SD	% missing data^*a*^	Mean ± SD	% missing data^*a*^	Mean ± SD	% missing data^*a*^	
DBP exposures (whole pregnancy)
Time-weighted average concentration (μg/L)
TTHM	45.6 ± 4.0	0	45.6 ± 4.1	0	45.6 ± 4.0	0	0.80
Chloroform	37.8 ± 3.8	0	37.6 ± 3.8	0	38.0 ± 3.7	0	< 0.001
BDCM	6.6 ± 0.6	0	6.7 ± 0.7	0	6.5 ± 0.5	0	< 0.001
DBCM	0.9 ± 0.2	0	0.9 ± 0.3	0	0.9 ± 0.2	0	< 0.001
THMBr	7.7 ± 0.8	0	7.8 ± 0.9	0	7.6 ± 0.6	0	< 0.001
DCAA	8.9 ± 2.1	15	8.5 ± 2.2	15	9.2 ± 2.0	15	< 0.001
TCAA	12.5 ± 2.6	15	12.5 ± 2.8	15	12.6 ± 2.4	15	0.17
BDCAA	1.3 ± 0.5	15	1.3 ± 0.5	15	1.3 ± 0.5	15	< 0.001
Integrated THM uptake (μg/day)
TTHM	1.86 ± 1.66	0	2.27 ± 1.98	0	1.49 ± 1.20	0	< 0.001
Chloroform	1.61 ± 1.46	0	1.96 ± 1.76	0	1.29 ± 1.05	0	< 0.001
BDCM	0.20 ± 0.16	0	0.24 ± 0.19	0	0.16 ± 0.13	0	< 0.001
DBCM	0.03 ± 0.03	0	0.04 ± 0.04	0	0.02 ± 0.02	0	< 0.001
THMBr	0.25 ± 0.21	0	0.30 ± 0.24	0	0.20 ± 0.16	0	< 0.001
Ingestion of HAA via drinking water consumption (μg/day)
DCAA	15.7 ± 10.4	15	17.5 ± 12.4	15	14.7 ± 8.3	15	< 0.001
TCAA	17.2 ± 11.0	15	18.5 ± 12.5	15	16.6 ± 9.2	15	< 0.001
BDCAA	1.6 ± 1.2	15	1.7 ± 1.3	15	1.7 ± 1.1	15	0.18
HAA3	34.5 ± 21.4	15	37.7 ± 24.7	15	32.9 ± 17.5	15	< 0.001
Abbreviations: BDCAA, bromodichloroacetic acid; BDCM, bromodichloromethane; DCAA, dichloroacetic acid; DBCM, dibromochloromethane; HAA3, the sum of DCAA, TCAA, and BDCAA; TCAA, trichloroacetic acid; THMBr, total brominated THMs; TTHM, total trihalomethanes. ^***a***^For 15% of women, time-weighted average HAA concentrations for their pregnancy could not be calculated because their pregnancy commenced before January 2007 (the earliest extent of the HAA models). ^***b***^*p*-Value for *t*-test and chi-square test for continuous and categorical variables respectively, comparing white British vs. Pakistani-origin.							

Table 3 presents adjusted model estimates for mean difference in birth weight associated with integrated TTHM and THMBr uptake tertiles for whole pregnancy, and trimester-specific results are shown in Supplemental Material, Table S3. Compared with bivariate models, adjusted coefficients changed considerably, indicating that the relationship between THM uptake and birth weight is confounded. For white British infants, there is no evidence of an association between birth weight and THM uptake. For Pakistani-origin infants, there are statistically significant reductions in mean birth weight of approximately 50 g for the highest exposure tertile compared with the lowest tertile [–53.7 g; 95% confidence interval (CI): –89.9, –17.5 for ≥ 1.82 vs. < 1.05 μg/day of total THM; and –56.4 g; 95% CI: –93.1, –19.6 for ≥ 0.26 vs. < 0.14 μg/day of THMBr] and significant trends (*p* < 0.01) across increasing exposure tertiles. Findings were similar for individual trimesters (see Supplemental Material, Table S3). *p*-Values for interaction by ethnicity were significant for TTHM uptake in whole pregnancy, and all THM uptakes in trimesters 2 and 3. Results for chloroform and BDCM uptake were similar (see Supplemental Material, Table S4).

**Table 3:** Relationship between term birth weight and whole pregnancy THM exposure.

Integrated THM uptake (μg/day)	*n*	Total (*n* = 7,438)		White British (*n* = 3,044)		Pakistani origin (*n* = 3,298)		*p*-Value interaction^*c*^
		Crude mean difference in term birth weight (g) (95% CI)	Adjusted^*a*^ mean difference in term birth weight (g) (95% CI)	*n*	Adjusted^*b*^ mean difference in term birth weight (g) (95% CI)	*n*	Adjusted^*b*^ mean difference in term birth weight (g) (95% CI)	
TTHM
< 1.05	2,538	Reference	Reference	750	Reference	1,432	Reference	0.011
≥ 1.05 to < 1.82	2,447	0.3 (–26.5, 27.0)	–20.1 (–43.0, 2.7)	980	–13.6 (–53.4, 26.1)	1,098	0.6 (–31.0, 32.1)
≥ 1.82	2,453	15.4 (–11.4, 42.2)	–21.6 (–45.3, 2.1)	1,344	10.8 (–26.9, 48.4)	768	–53.7 (–89.9, –17.5)
*p*-Value for trend^*d*^		0.262	0.072		0.442		0.009
*p*-Value for significance^*e*^		0.438	0.125		0.371		0.006
THMBr
< 0.14	2,516	Reference	Reference	678	Reference	1,491	Reference	0.074
≥ 0.14 to < 0.26	2,496	20.1 (–6.6, 46.8)	–11.0 (–33.9, 11.8)	1,000	1.6 (–38.8, 42.0)	1,086	–6.5 (–38.0, 25.0)
≥ 0.26	2,426	27.7 (0.8, 54.6)	–20.0 (–44.0, 3.9)	1,366	6.5 (–31.9, 45.0)	721	–56.4 (–93.1, –19.6)
*p*-Value for trend^*d*^		0.043	0.101		0.717		0.006
*p*-Value for significance^*e*^		0.112	0.256		0.070		0.007
Abbreviations: BDCM, bromodichloromethane; THMBr, total brominated THMs; TTHM, total trihalomethanes. ^***a***^Adjusted for 10 maternal factors (caffeine intake, IMD, education, fasting glucose, post-load glucose, ethnicity, smoking, parity, age, BMI) and 2 infant factors (gestational age at delivery as linear and quadratic terms, sex). ^***b***^Ethnic subgroup analyses excluded ethnicity covariate. ^***c***^*p*-Value for significance of exposure–ethnicity interaction term, as a whole, from *F*-test. ^***d***^*p*-Value for linear trend across tertiles, derived by including the exposure term (coded as 0, 1, 2) as continuous variable in the model. ^***e***^*p*-Value for significance of categorical exposure term, as a whole, within the model, from *F*-test.								

We find no evidence of association between birth weight and HAA ingestion (via drinking water consumption) for either the total population or ethnic subgroups for whole pregnancy (Table 4) or for separate trimesters (data not shown).

**Table 4:** Relationship between term birth weight and whole pregnancy HAA ingestion.

Ingestion (μg/day)	*n*	Total (*n* = 6,529)		White British (*n* = 2,651)		Pakistani origin (*n* = 2,916)		*p*-Value interaction^*c*^
		Crude mean difference in term birth weight (g) (95% CI)	Adjusted^*a*^ mean difference in term birth weight (g) (95% CI)	*n*	Adjusted^*b*^ mean difference in term birth weight (g) (95% CI)	*n*	Adjusted^*b*^ mean difference in term birth weight (g) (95% CI)	
BDCAA
< 1.01	2,247	Reference	Reference	930	Reference	899	Reference	0.876
≥ 1.01 to < 1.86	2,145	–7.0 (–35.6, 21.5)	0.7 (–23.3, 24.8)	835	4.3 (–34.0, 42.6)	1,030	14.2 (–21.3, 49.7)
≥ 1.86	2,137	–13.4 (–42.0, 15.1)	1.6 (–22.9, 26.0)	886	19.2 (–19.5, 57.9)	987	19.0 (–17.5, 5.5)
*p*-Value for trend^*d*^		0.357	0.901		0.331		0.310
*p*-Value for significance^*e*^		0.654	0.992		0.590		0.568
HAA3
< 23.82	2,132	Reference	Reference	843	Reference	895	Reference	0.971
≥ 23.82 to < 38.83	2,174	–34.9 (–63.7, –6.1)	–18.9 (–43.3, 5.6)	771	–9.5 (–50.4, 31.3)	1,120	0.1 (–34.9, 35.1)
≥ 38.83	2,223	–11.6 (–40.3, 17.0)	–0.6 (–25.5, 24.4)	1,037	11.7 (–27.5, 50.9)	901	13.1 (–24.8, 51.0)
*p*-Value for trend^*d*^		0.443	0.973		0.525		0.498
*p*-Value for significance^*e*^		0.053	0.222		0.558		0.721
Abbreviations: BDCAA, bromodichloroacetic acid; DCAA, dichloroacetic acid; HAA, haloacetic acid; HAA3, sum of DCAA, TCAA, and BDCAA; TCAA, trichloroacetic acid. ^***a***^Adjusted for 10 maternal factors (caffeine intake, IMD, education, fasting glucose, post-load glucose, ethnicity, smoking, parity, age, BMI) and 2 infant factors (gestational age at delivery as linear and quadratic terms, sex). ^***b***^Ethnic subgroup analyses excluded ethnicity covariate. ^***c***^*p*-Value for significance of exposure–ethnicity interaction term, as a whole, from *F*-test. ^***d***^*p*-Value for linear trend across tertiles, derived by including the exposure term (coded as 0, 1, 2) as continuous variable in the model. ^***e***^*p*-Value for significance of categorical exposure term, as a whole, within the model, from *F*-test.								

In joint THM and HAA exposure models (Table 5; see also Supplemental Material, Table S5), we find no evidence of association between birth weight and DBP7 ingestion via water consumption. However, for TTHM uptake via showering, bathing, and swimming we observe statistically significant trends across tertiles, and reductions in mean birth weight for the highest exposure tertile for Pakistani-origin infants for whole-pregnancy (–67.4 g; 95% CI: –106.1, –28.6) and trimester-specific exposures. Interactions between ethnicity and TTHM uptake via showering, bathing, and swimming were significant except for trimester 1. We found statistically significant trends across time-weighted average DBP7 concentration tertiles for whole pregnancy (mean birth weight difference for highest tertile: –60.2 g; 95% CI: –117.4, –3.1) (Table 5) for Pakistani-origin infants, and for white British infants for trimester 1 (trimester-specific data not shown).

**Table 5:** Relationship between term birth weight and whole pregnancy joint THM and HAA exposures (DBP7).

Exposure	Total (*n* = 6,529)			White British (*n *= 2,651)		Pakistani origin (*n *= 2,916)		*p*-Value interaction^*c*^
	*n*	Crude mean difference in term birth weight (g) (95% CI)	Adjusted^*a*^ mean difference in term birth weight (g) (95% CI)	*n*	Adjusted^*b*^ mean difference in term birth weight (g) (95% CI)	*n*	Adjusted^*b*^ mean difference in term birth weight (g) (95% CI)	
Individual‑level exposure model including 2 exposure terms^*d*^
DBP7 via drinking water consumption (μg/day)^*e*^
< 58.6	2,162	Reference	Reference	957	Reference	817	Reference	0.640
≥ 58.6–97.0	2,173	–39.4 (–68.2, –10.7)	–23.6 (–48.0, 0.8)	795	–16.3 (–55.3, 22.7)	1,090	4.8 (–31.8, 41.5)
≥ 97.0	2,194	–14.5 (–43.2, 14.1)	0.4 (–24.0, 24.8)	899	21.3 (–16.4, 58.9)	1,009	18.1 (–19.5, 55.8)
*p*-Value for trend^*f*^		0.326	0.955		0.278		0.335
*p*-Value for significance^*g*^		0.025	0.085		0.171		0.595
Uptake of TTHM via showering, bathing, swimming (μg/day)^*h*^
< 0.85	2,228	Reference	Reference	626	Reference	1,308	Reference	0.032
≥ 0.85–1.63	2,148	3.8 (–24.8, 32.4)	–23.9 (–48.2, 0.5)	844	–28.9 (–71.9, 14.2)	938	–14.9 (–48.7, 18.9)
≥ 1.63	2,153	11.2 (–17.4, 39.8)	–31.7 (–57.0, –6.4)	1,181	–8.2 (–48.5, 32.1)	670	–67.4 (–106.1, –28.6)
*p*-Value for trend^*f*^		0.371	0.017		0.912		0.001
*p*-Value for significance^*g*^		0.739	0.036		0.361		0.002
Time-weighted average DBP7 concentration (μg/L)^*i*^
< 65	1,426	Reference	Reference	649	Reference	555	Reference	0.589
≥ 65–75	4,410	–43.3 (–72.0, –14.5)	–30.7 (–54.9, –6.5)	1,729	–30.7 (–66.6, 5.3)	2,041	–35.2 (–73.1, 2.7)
≥ 75	693	–99.8 (–143.5, –56.1)	–44.8 (–82.3, –7.4)	273	–20.1 (–78.6, 38.3)	320	–60.2 (–117.4, –3.1)
*p*-Value for trend^*f*^		< 0.001	0.007		0.251		0.027
*p*-Value for significance^*g*^		< 0.001	0.020		0.277		0.073
Abbreviations: DBP7, sum of TTHM, DCAA, TCAA, and BDCAA; HAA, haloacetic acid; THM, trihalomethanes; TTHM, total trihalomethanes. ^***a***^Adjusted for 10 maternal factors (caffeine intake, IMD, education, fasting glucose, post-load glucose, ethnicity, smoking, parity, age, BMI) and 2 infant factors (gestational age at delivery as linear and quadratic terms, sex). ^***b***^Ethnic subgroup analyses excluded ethnicity covariate. ^***c***^*p*-Value for significance of exposure–ethnicity interaction term, as a whole, from *F*-test. ^***d***^This model includes both exposure terms: DBP7 via drinking water consumption; and uptake of TTHM via showering, bathing, swimming. ^***e***^Consumption via drinking water (μg/day) of sum of TTHM, DCAA, TCAA, and BDCAA. ^***f***^*p*-Value for linear trend across tertiles, derived by including the exposure term (coded as 0, 1, 2) as continuous variable in the model. ^***g***^*p*-Value for significance of categorical exposure term, as a whole, within the model, from *F*-test. ^***h***^μg/day uptake into blood via these activities. ^***i***^Sum of time-weighted average concentrations of TTHM, DCAA, TCAA, and BDCAA.								

Longer bathing duration is associated with birth weight reductions for Pakistani-origin but not white British infants (Pakistani-origin: –55.9 g; 95% CI: –102.2, –9.7 and white British: –17.6 g; 95% CI: –59.3, 24.2 for > 120 min/week vs. 0 min/week bathing). Cold tap water consumption is associated with increased birth weight for Pakistani-origin infants but not for white British infants (Pakistani-origin: 71.4 g; 95% CI: 23.4, 119.4 and white British: 23.7 g; 95% CI: –16.6, 63.9 for ≥ 1.6 L/day vs. 0–0.4 L/day cold tap water consumption).

We examined differences between Pakistani-origin women in the highest THM uptake tertile versus those in the lower tertiles. Those in the highest tertile were slightly younger (highest tertile: mean age, 27.0 years; 95% CI: 26.7, 27.4 vs. lower tertiles: mean age, 27.7 years; 95% CI: 27.6, 27.8), 22% versus 42% completed the questionnaire in Urdu or Mirpuri, 28% versus 21% were employed, 8% versus 12% had gestational diabetes. In terms of lifestyle, 6% versus 2% were current smokers, 33% versus 23% were exposed to environmental tobacco smoke, and 7% versus 5% consumed ≥ 200 mg caffeine per day. In sensitivity analyses additionally adjusting models for environmental tobacco smoke and language of questionnaire completion, neither variable altered the relationship between birth weight and THM uptake for Pakistani-origin infants (data not shown).

## Discussion

To our knowledge, this is by far the largest study to date on DBPs and birth weight with data on individual-level water use. Moreover, it is the first study in DBP epidemiology to incorporate detailed individual-level estimates of joint THM–HAA exposures. Our results suggest that maternal exposure to THMs during pregnancy (measured as both integrated THM uptake and exposure to THMs via showering, bathing, and swimming) is inversely associated with term birth weight for infants of Pakistani-origin. No associations were observed for white British infants. We found no evidence of association between birth weight and ingestion of HAAs alone, or THMs and HAAs combined, via drinking water consumption.

Strengths of our exposure assessment were modeling of DBP data to give estimates both temporally and spatially, even for times and places for which data were sparse; comprehensive exposure assessment combining individual-level water usage with modeled area-level DBP concentrations; and accounting for exposure modifiers (boiling/filtering).

Given the size of our study, it has greater statistical power than similar studies that have preceded it. It also benefits from many detailed individual-level covariates to address potential confounding and the homogeneity of the Pakistani-origin ethnic classification, because we did not lump all South Asian ethnicities together.

It is a limitation that we could not account for residential mobility during pregnancy, and only partially account for mobility between WSZs due to workplace location. However, given the low spatial variability in DBPs between WSZs in the study area, we expect the impact to be small in terms of exposure misclassification.

Compared with previous studies that had similar exposure assessment (i.e., integrated THM uptake), the 50-g birth weight reduction in Pakistani-origin infants we estimated for TTHM uptake > 1.82 μg/day (compared with reference < 1.05 μg/day, representing an exposure contrast of at least 0.77 μg/day) is consistent with 45- to 50-g birth weight reduction associated with a 1-μg/day increase in TTHM internal dose reported by Grazuleviciene et al. (2011). In contrast, two previous studies report no evidence for an association between birth weight and personal THM exposure, either as integrated THM uptake or via separate consumption or showering/bathing pathways (Hoffman et al. 2008; Villanueva et al. 2011).

We found no evidence of association between HAA exposure and birth weight, consistent with three other studies (Hoffman et al. 2008; Horton et al. 2011; Wright et al. 2004). In contrast, other studies have reported birth weight deficits associated with HAA5 exposure (Rivera-Núñez and Wright 2013) and high levels of urinary TCAA (Zhou et al. 2012).

We found evidence of association between time-weighted average DBP7 concentration and reduced birth weight, similar to the results of Rivera-Núñez and Wright (2013), who reported birth weight deficits of 39–45 g for DBP9 (sum of TTHM and HAA5) concentration (e.g., –39 g; 95% CI: –62, –16 for > 97 μg/L vs. 0 μg/L of DBP9). However, we found no evidence of association for individual-level DBP7 ingestion. This may reflect the positive health benefits of higher water consumption, and that women who drink lots of water tend to be healthier, both of which could mask possible adverse effects of DBP exposure via this route; and we did observe associations between cold tap water consumption and increased birth weight. Behavior in pregnancy may be influenced by the health of the pregnancy, and we cannot rule out reverse causality in the water consumption or bathing associations we observe. That we observed a relationship between birth weight and DBP7 levels in tap water suggests that the relationship between integrated THM exposure and birth weight observed for Pakistani-origin infants cannot be explained by residual confounding due to unmeasured variables related to showering and bathing (e.g., use of personal care products, which could differ by ethnicity).

We observed that uptake of TTHM via showering, bathing, and swimming—predominantly via inhalation and dermal absorption routes—was associated with reduced birth weight in a dose–response manner for Pakistani-origin infants, when adjusting for exposure to THM and HAA ingestion via water consumption. This suggests that birth weight reductions observed in integrated THM uptake models are driven by exposure via showering, bathing, and swimming (rather than consumption). A previous study also found THM exposure via showering/bathing, but not other routes, to be associated with fetal growth restriction (Costet et al. 2012).

To explain inconsistent findings in DBP epidemiology, it has been hypothesized that THMs may be a proxy for an unmeasured putative agent, for example, another DBP/pollutant. The present study suggests that THMs are not acting as a proxy for HAAs, or vice versa. It is possible that the association we observed between birth weight and THM uptake could reflect other DBPs (e.g., emerging DBPs), for which THMs could act as a proxy. However, of 24 previous studies examining quantitative measures of THM exposure and fetal growth outcomes, 18 report evidence for associations between exposure and outcome (Bove et al. 1995; Costet et al. 2012; Danileviciute et al. 2012; Gallagher et al. 1998; Grazuleviciene et al. 2011; Hinckley et al. 2005; Hoffman et al. 2008; Iszatt et al. 2014; Kramer et al. 1992; Kumar et al. 2014; Levallois et al. 2012; Lewis et al. 2006; Porter et al. 2005; Rivera-Núñez and Wright 2013; Summerhayes et al. 2012; Toledano et al. 2005; Wright et al. 2003, 2004), whereas 6 do not (Dodds et al. 1999; Horton et al. 2011; Patelarou et al. 2011; Savitz et al. 1995; Villanueva et al. 2011; Yang et al. 2007). It is thus difficult to dismiss THMs as a proxy for other DBPs, because it would have to be the same proxy in all these studies. This is unlikely, given that DBP mixture composition and corresponding toxicity has been shown to vary substantially in different study locations (Jeong et al. 2012).

To our knowledge, there is no existing literature on the relationship between birth weight and DBP exposure specifically for infants of Pakistani-origin with which we can compare our main findings. However, ethnic differences (Caucasian vs. non-Caucasian) in term LBW risk associated with high TTHM exposure have previously been observed (Lewis et al. 2006).

Smoking, drinking alcohol, and caffeine consumption are known risk factors for adverse fetal growth outcomes, which may also be related to water consumption/activities and thus potentially confound the relationship between DBP exposure and birth weight. In the present study, very few Pakistani-origin women smoked, drank alcohol, or consumed ≥ 200 mg caffeine per day, but prevalence of these activities was much higher among white British women. With higher prevalence, these risk factors may have greater impact in the white British subpopulation—the exposure–outcome relationship could be more confounded, or the adverse effects of these risk factors may dwarf potential deleterious effects of DBPs, making it more difficult to tease out effects of DBPs in this population. The differences in effect between ethnic groups could be explained by residual confounding because other factors such as diet and stress, which may differ by ethnicity, were not taken into account in this study.

Alternatively, there may be potential differences in DBP uptake or metabolism by ethnicity, resulting from differences in body composition, hepatic function, or genetic variation between ethnic groups. South Asian adults (including those of Pakistani origin) have greater relative fat mass than European-origin adults (McKeigue et al. 1991). Additionally, in this population Pakistani-origin infants, although lighter in weight, are relatively more adipose than white British infants (West et al. 2013). THMs take longer to partition out of physiological compartments such as adipose tissue, compared to blood (Blount et al. 2011). Greater fat mass could result in greater relative uptake of THMs, which are lipophilic, into adipose tissue for the same maternal exposure, potentially resulting in longer-lived exposure for those with greater fat mass.

It is also plausible that ethnic variation in genes related to metabolic enzymes could result in different patterns of metabolism by ethnicity. Specific polymorphisms in *CYP2D6* and *GSTT1* genes have been associated with significant differences in blood THM concentrations following showering (Backer et al. 2008). Other chemical compounds demonstrate different patterns of metabolism by ethnicity—for example, arsenic (Brima et al. 2006) and various pharmaceutical drugs (Yasuda et al. 2008). Within BiB, associations between residential greenness and birth weight differ by ethnicity, with a positive association for white British infants, but not for those of Pakistani-origin (Dadvand et al. 2014).

## Conclusions

This large study, which is the first to incorporate individual-level ingestion estimates of DBP mixtures, substantially strengthens a considerable body of evidence suggesting that exposure to THMs during pregnancy is associated with adverse fetal growth outcomes, including reduced birth weight. Our observation of a birth weight reduction of approximately 50 g associated with high THM exposure for Pakistani-origin infants could have a proportionally greater health impact on these infants now and throughout life, because they already weigh considerably less on average than white British infants at birth. This study makes a valuable contribution to the more limited evidence base for HAAs by indicating that HAA exposures are not associated with term birth weight in our study population.

There is virtually no epidemiological literature on THM–HAA mixtures and fetal growth, and this study advances the field in this respect by showing that associations with THMs were not the result of confounding by HAA exposures in our study population. Any future studies of DBPs and fetal growth should examine as wide a range of DBP classes/species as possible, and assess mixture effects. Further studies examining only single classes of DBPs are not warranted because they are unlikely to advance this field.

## Supplemental Material

(343 KB) PDF
