# Supplementary material for: Birth Weight, Ethnicity, and Exposure to Trihalomethanes and Haloacetic Acids in Drinking Water during Pregnancy in the Born in Bradford Cohort
Source: Environ Health Perspect. 2015 Sep 4;124(5):681–9. doi: 10.1289/ehp.1409480 (PMC4858386; doi:10.1289/ehp.1409480)
Supplement: (343 KB) PDF [file ehp.1409480.s001.acco.pdf]

Environ Health Perspect
DOI: 10.1289/ehp.1409480

Note to Readers: *EHP* strives to ensure that all journal content is accessible to all readers. However, some figures and Supplemental Material published in *EHP* articles may not conform to 508 standards due to the complexity of the information being presented. If you need assistance accessing journal content, please contact ehp508@niehs.nih.gov. Our staff will work with you to assess and meet your accessibility needs within 3 working days.

Supplemental Material

Birth Weight, Ethnicity, and Exposure to Trihalomethanes and Haloacetic Acids in Drinking Water during Pregnancy in the Born in Bradford Cohort

Rachel B. Smith, Susan C. Edwards, Nicky Best, John Wright, Mark J. Nieuwenhuijsen, and Mireille B. Toledano

Table of Contents

THM uptake factors
Table S1. THM uptake factors
References
Table S2. Spearman's correlations between exposure metrics used in epidemiological models
Table S3. Relationship between term birth weight and TTHM and THMBr exposure in Trimester 1, 2 and 3
Table S4. Relationship between term birth weight and chloroform and BDCM exposure
Table S5. Relationship between term birth weight and individual-level joint THM and HAA exposure (DBP7) in Trimester 1, 2 and 3

THM uptake factors

Uptake factor parameter values used are shown in Table S1. THM uptake factors were calculated from biomonitoring studies (Aggazzotti et al. 1995; Backer et al. 2000; Lynberg et al. 2001) which had measured blood or plasma THM concentrations before and after ingestion of THM-containing water or showering, bathing or swimming in THM-containing water, and had measured THM concentrations in tap or swimming pool water. The uptake factors were calculated as per the examples below for chloroform, based on methodology by Whitaker *et al*. (2003) where possible, or where necessary based on uptake factors previously used in the literature.

Ingestion uptake factor - Uptake (per litre of water ingested per μg/l chloroform) is given by the proportion of chloroform in the blood to the total amount of chloroform ingested:

$$\frac{[\text{Blood volume (l)} * (\text{Blood chloroform concentration after ingestion } (\mu g/l) - \text{Blood chloroform concentration before ingestion } (\mu g/l))]}{\text{Water chloroform concentration } (\mu g/l) * \text{Volume water ingested (l)}}$$

Showering uptake factor - Uptake (per μg/l chloroform in water per minute spent showering) is given by:

$$\frac{[\text{Blood volume (l)} * (\text{Blood chloroform concentration after showering } (\mu g/l) - \text{Blood chloroform concentration before showering } (\mu g/l))]}{\text{Water THM concentration } (\mu g/l) * \text{Duration of showering (min)}}$$

Bathing uptake factor: as per formula for showering.

Swimming uptake factor - Uptake (per μg/l chloroform in water per minute spent in the swimming pool) is given by:

$$\frac{[\text{Blood volume (l)} * (\text{Plasma chloroform concentration after swim } (\mu g/l) - \text{Plasma chloroform concentration before swim } (\mu g/l)) * 2.135]}{\text{Water THM concentration } (\mu g/l) * \text{Duration of swim (min)}}$$

The formulae assume a whole body blood volume of 5 litres. For the swimming uptake factor 2.135 represents a plasma correction factor. If the uptake factors for a particular activity were calculated from more than one study, then a weighted average of uptake factors from all the studies were taken based upon study size.

Table S1. THM uptake factors

Activity	DBP	Units	Uptake factor	Reference
**Ingestion**	Chloroform	µg/(µg/l)l	0.00490196	Backer *et al.*(2000)
	DCBM	µg/(µg/l)l	0.00108696	Backer *et al.* (2000)
	DBCM	µg/(µg/l)l	0.00115	Backer *et al.* (2000)
	Brominated THM	µg/(µg/l)l	0.00111848	Average of DCBM and DBCM uptake factors above (as used by Villanueva *et al.*(2007))
**Showering**	Chloroform	µg/(µg/l)min	0.001563091	Weighted average from Backer *et al.* (2000) and Lynberg *et al.* (2001)
	DCBM	µg/(µg/l)min	0.001322253	Weighted average from Backer *et al.* (2000) and Lynberg *et al.* (2001)
	DBCM	µg/(µg/l)min	0.001355042	Weighted average from Backer *et al.* (2000) and Lynberg *et al.* (2001)
	Brominated THM	µg/(µg/l)min	0.0013386475	Average of DCBM and DBCM uptake factors for showering above.
**Bathing**	Chloroform	µg/(µg/l)min	0.001320755	Backer *et al.* (2000)
	DCBM	µg/(µg/l)min	0.001189711	Backer *et al.* (2000)
	DBCM	µg/(µg/l)min	0.001401709	Backer *et al.* (2000)
	Brominated THM	µg/(µg/l)min	0.00129571	Average of DCBM and DBCM uptake factors for bathing above.
**Swimming**	Chloroform	µg/(µg/l)min	0.002541407	Aggazzotti *et al.*(1995) – based on non-competitive swimmers.
**Swimming**	Brominated THM	µg/(µg/l)min	0.0022367211	As used by Villanueva *et al.* (2007) – based on expert judgement.

References

Aggazzotti G, Fantuzzi G, Righi E, Predieri G. 1995. Environmental and biological monitoring of chloroform in indoor swimming pools. Journal of Chromatography A 710:181.

Backer LC, Ashley DL, Bonin MA, Cardinali FL, Kieszak SM, Wooten JV. 2000. Household exposures to drinking water disinfection by-products: whole blood trihalomethane levels. J Expo Anal Environ Epidemiol 10:321-326.

Lynberg M, Nuckols JR, Langlois P, Ashley D, Singer P, Mendola P, et al. 2001. Assessing exposure to disinfection by-products in women of reproductive age living in Corpus Christi, Texas, and Cobb county, Georgia: descriptive results and methods. Environ Health Perspect 109:597-604.

Villanueva CM, Gagniere B, Monfort C, Nieuwenhuijsen MJ, Cordier S. 2007. Sources of variability in levels and exposure to trihalomethanes. Environ Res 103:211-220.

Whitaker HJ, Nieuwenhuijsen MJ, Best NG. 2003. The relationship between water concentrations and individual uptake of chloroform: a simulation study. Environ Health Perspect 111:688-694.

Table S2. Spearman's correlations between exposure metrics used in epidemiological models

	TTHM uptake (WP)	TTHM uptake (T1)	TTHM uptake (T2)	TTHM uptake (T3)	Chloroform uptake (WP)	DCBM uptake (WP)	THMBr uptake (WP)	DCAA ingestion (WP)	TCAA ingestion (WP)	BDCAA ingestion (WP)	HAA3 ingestion (WP)	THM & HAA via consumption (WP)	TTHM uptake via shower/bath/swim (WP)	Cold Tap Water (L/day)	Total Tap Water (L/day)	Total Water (L/day)	Showering (min/wk)	Bathing (min/wk)	Combined showering/bathing (min/wk)	Swimming (min/wk)	TW-ave DBP7 (WP)
**TTHM uptake (WP)**	1.00																				
**TTHM uptake (T1)**	0.93	1.00																			
**TTHM uptake (T2)**	0.97	0.87	1.00																		
**TTHM uptake (T3)**	0.93	0.77	0.88	1.00																	
**Chloroform uptake (WP)**	1.00	0.93	0.97	0.93	1.00																
**DCBM uptake (WP)**	0.98	0.92	0.93	0.91	0.97	1.00															
**THMBr uptake (WP)**	0.98	0.92	0.93	0.91	0.97	1.00	1.00														
**DCAA ingestion (WP)**	0.14	0.14	0.12	0.14	0.15	0.05	0.05	1.00													
**TCAA ingestion (WP)**	0.17	0.15	0.16	0.18	0.19	0.06	0.06	0.85	1.00												
**BDCAA ingestion (WP)**	0.15	0.14	0.14	0.15	0.16	0.06	0.06	0.72	0.86	1.00											
**HAA3 ingestion (WP)**	0.16	0.15	0.15	0.16	0.18	0.06	0.06	0.95	0.97	0.84	1.00										
**THM & HAA via consumption (WP)**	0.17	0.15	0.17	0.16	0.19	0.06	0.05	0.80	0.94	0.87	0.91	1.00									
**TTHM uptake via shower/bath/swim (WP)**	0.98	0.92	0.95	0.92	0.98	0.99	0.98	0.03	0.04	0.02	0.04	0.02	1.00								
**Cold Tap Water (L/day)**	0.13	0.12	0.12	0.13	0.15	0.03	0.03	0.59	0.76	0.71	0.71	0.86	-0.01	1.00							
**Total Tap Water (L/day)**	0.18	0.16	0.17	0.17	0.19	0.10	0.10	0.85	0.86	0.74	0.89	0.85	0.06	0.79	1.00						
**Total Water (L/day)**	0.23	0.21	0.22	0.22	0.24	0.17	0.17	0.68	0.67	0.55	0.70	0.64	0.14	0.58	0.81	1.00					
**Showering (min/wk)**	0.38	0.34	0.36	0.37	0.38	0.38	0.35	-0.06	-0.03	-0.04	-0.05	-0.04	0.39	0.00	-0.03	0.04	1.00				
**Bathing (min/wk)**	0.49	0.48	0.46	0.45	0.48	0.54	0.56	0.10	0.06	0.04	0.08	0.04	0.50	0.00	0.07	0.08	-0.39	1.00			
**Combined showering/bathing (min/wk)**	0.87	0.82	0.81	0.81	0.86	0.93	0.92	0.04	0.03	0.01	0.04	0.01	0.89	-0.01	0.05	0.11	0.34	0.64	1.00		
**Swimming (min/wk)**	0.42	0.41	0.41	0.41	0.42	0.33	0.35	0.02	0.03	0.01	0.02	0.02	0.42	0.02	0.05	0.12	0.06	0.01	0.05	1.00	
**TW-ave DBP7 (WP)**	0.09	0.04	0.19	0.04	0.11	0.02	0.01	0.12	0.22	0.23	0.18	0.12	0.08	-0.01	0.00	-0.02	0.00	0.00	0.01	-0.02	1.00

Abbreviations: WP, whole pregnancy average; TW-ave, time-weighted average concentration. Red cells = Spearman's correlation ≥ 0.8, green cells = Spearman's correlation ≥ 0.5 to < 0.8.

Table S3. Relationship between term birth weight and TTHM and THMBr exposure in Trimester 1, 2 and 3

Time window	Integrated THM uptake (µg/day)	TOTAL (n=7438) N	TOTAL (n=7438) Adjusted[a] mean difference in term birth weight (g)(95% CI)	WHITE BRITISH (n=3044) N	WHITE BRITISH (n=3044) Adjusted[b] mean difference in term birth weight (g)(95% CI)	PAKISTANI ORIGIN (n=3298) N	PAKISTANI ORIGIN (n=3298) Adjusted[b] mean difference in term birth weight (g)(95% CI)	p for interaction[c]
**Trimester 1**	***TTHM***							
	**< 1.05**	2644	Reference	798	Reference	1476	Reference	0.087
	**≥1.05 - <1.82**	2319	-25.8 (-48.4, -3.1)	898	9.3 (-29.7, 48.4)	1052	-20.9 (-52.7, 10.9)	
	**≥1.82**	2475	-17.4 (-40.9, 6.0)	1348	18.4 (-18.6, 55.4)	770	-42.7 (-78.7, -6.7)	
	p for trend[d]		0.133		0.324		0.018	
	p for significance[e]		0.077		0.604		0.056	
**Trimester 2**	***TTHM***							
	**< 1.05**	2656	Reference	801	Reference	1498	Reference	0.006
	**≥1.05 - <1.82**	2344	-2.0 (-24.9, 20.8)	916	-25.6 (-64.6, 13.5)	1029	20.4 (-11.6, 52.4)	
	**≥1.82**	2438	-18.4 (-41.5, 4.6)	1327	-1.3 (-37.2, 34.6)	771	-41.9 (-77.3, -6.6)	
	p for trend[d]		0.122		0.879		0.063	
	p for significance[e]		0.238		0.309		0.005	
**Trimester 3**	***TTHM***							
	**< 1.05**	2728	Reference	826	Reference	1504	Reference	0.003
	**≥1.05 - <1.82**	2264	-10.3 (33.2, 12.7)	898	-20.2 (-59.4, 19.1)	1004	11.6 (-20.4, 43.7)	
	**≥1.82**	2446	-7.7 (-30.8, 15.4)	1320	13.6 (-22.8, 50.0)	790	-46.9 (-82.4, -11.3)	
	p for trend[d]		0.500		0.344		0.026	
	p for significance[e]		0.654		0.162		0.006	
**Trimester 1**	***THMBr***							
	**< 0.14**	2472	Reference	666	Reference	1474	Reference	0.113
	**≥0.14 - <0.26**	2548	-24.5 (-47.3, -1.7)	1018	-4.5 (-44.8, 35.7)	1100	-19.1 (-50.5, 12.3)	
	**≥0.26**	2418	-21.6 (-45.7, 2.5)	1360	8.1 (-30.3, 46.6)	1360	-51.7 (-88.8, -14.5)	
	p for trend[d]		0.077		0.593		0.007	
	p for significance[e]		0.079		0.751		0.020	

Time window	Integrated THM uptake (µg/day)	N	TOTAL (n=7438) Adjusted[a] mean difference in term birth weight (g)(95% CI)	N	WHITE BRITISH (n=3044) Adjusted[b] mean difference in term birth weight (g)(95% CI)	N	PAKISTANI ORIGIN (n=3298) Adjusted[b] mean difference in term birth weight (g)(95% CI)	p for interaction[c]
**Trimester 2**	***THMBr***							
	**< 0.14**	2528	Reference	686	Reference	1490	Reference	0.039
	**≥0.14 - <0.26**	2515	-8.3 (-31.1, 14.6)	1019	-2.2 (-42.4, 37.9)	1089	0.4 (-31.3, 32.1)	
	**≥0.26**	2395	-20.2 (-43.9, 3.5)	1339	6.9 (-31.2, 44.9)	719	-56.3 (-92.7, -19.9)	
	p for trend[d]		0.096		0.667		0.007	
	p for significance[e]		0.249		0.857		0.004	
**Trimester 3**	***THMBr***							
	**< 0.14**	2601	Reference	711	Reference	1513	Reference	0.044
	**≥0.14 - <0.26**	2448	-10.9 (-33.7, 11.9)	996	1.2 (-38.7, 41.0)	1063	-7.5 (-39.0, 24.1)	
	**≥0.26**	2389	-12.4 (-36.0, 11.2)	1337	14.4 (-23.3, 52.0)	722	-52.8 (-89.3, -16.3)	
	p for trend[d]		0.295		0.407		0.009	
	p for significance[e]		0.520		0.664		0.014	

Abbreviations: BDCM, bromodichloromethane; THMBr, total brominated THMs; TTHM, total trihalomethanes. [a]Adjusted for 10 maternal factors (caffeine intake, IMD, education, fasting glucose, post load glucose, ethnicity, smoking, parity, age, BMI) and 2 infant factors (gestational age at delivery as linear and quadratic terms, sex). [b]Ethnic sub-group analyses excluded ethnicity covariate. [c]p-value for significance of exposure-ethnicity interaction term, as a whole, from F-test. [d]p-value for linear trend across tertiles, derived by including the exposure term (coded as 0, 1, 2) as continuous variable in the model. [e]p-value for significance of categorical exposure term, as a whole, within the model, from F-test.

Table S4. Relationship between term birth weight and chloroform and BDCM exposure

Time window	Integrated THM uptake (µg/day)	TOTAL (n=7438) N	TOTAL (n=7438) Adjusted[a] mean difference in term birth weight (g)(95% CI)	WHITE BRITISH (n=3044) N	WHITE BRITISH (n=3044) Adjusted[b] mean difference in term birth weight (g)(95% CI)	PAKISTANI ORIGIN (n=3298) N	PAKISTANI ORIGIN (n=3298) Adjusted[b] mean difference in term birth weight (g)(95% CI)	p for interaction[c]
**Whole pregnancy**	***Chloroform***							
	**< 0.91**	2538	Reference	760	Reference	1421	Reference	0.011
	**≥0.91 - <1.56**	2463	-16.3 (-39.0, 6.5)	952	-13.3 (-52.9, 26.3)	1110	10.3 (-21.2, 41.9)	
	**≥1.56**	2437	-20.9 (-44.6, 2.8)	1332	9.0 (-28.5, 46.5)	767	-48.3 (-84.6, -12.1)	
	p for trend[d]		0.082		0.510		0.025	
	p for significance[e]		0.181		0.436		0.006	
**Trimester 1**	***Chloroform***							
	**< 0.91**	2707	Reference	838	Reference	1488	Reference	0.094
	**≥0.91 - <1.56**	2249	-26.5 (-49.3, -3.8)	866	9.4 (-29.5, 48.3)	1035	-18.3 (-50.2, 13.5)	
	**≥1.56**	2482	-17.2 (-40.4, 6.0)	1340	16.1 (-20.2, 52.5)	775	-43.4 (-79.2, -7.6)	
	p for trend[d]		0.131		0.385		0.017	
	p for significance[e]		0.067		0.677		0.054	
**Trimester 2**	***Chloroform***							
	**< 0.91**	2710	Reference	829	Reference	1510	Reference	0.016
	**≥0.91 - <1.56**	2291	-1.4 (-24.4, 21.5)	901	-18.7 (-57.4, 20.0)	1011	17.9 (-14.5, 50.3)	
	**≥1.56**	2437	-19.8 (-42.6, 3.0)	1314	0.7 (-34.6, 36.0)	777	-42.0 (-77.1, -6.9)	
	p for trend[d]		0.094		0.845		0.056	
	p for significance[e]		0.179		0.498		0.007	
**Trimester 3**	***Chloroform***							
	**< 0.91**	2760	Reference	855	Reference	1497	Reference	0.007
	**≥0.91 - <1.56**	2238	-14.8 (-37.7, 8.1)	877	-27.0 (-66.1, 12.1)	1003	5.1 (-27.1, 37.4)	
	**≥1.56**	2440	-8.7 (-31.8, 14.3)	1312	9.5 (-26.8, 45.8)	798	-42.8 (-78.2, -7.4)	
	p for trend[d]		0.435		0.461		0.035	
	p for significance[e]		0.440		0.117		0.023	
**Whole pregnancy**	***BDCM***							
	**< 0.12**	2668	Reference	744	Reference	1558	Reference	0.111

Time window	Integrated THM uptake (µg/day)	N	TOTAL (n=7438) Adjusted[a] mean difference in term birth weight (g)(95% CI)	N	WHITE BRITISH (n=3044) Adjusted[b] mean difference in term birth weight (g)(95% CI)	N	PAKISTANI ORIGIN (n=3298) Adjusted[b] mean difference in term birth weight (g)(95% CI)	p for interaction[c]
	**≥0.12 - <0.21**	2348	-11.1 (-33.9, 11.8)	947	8.2 (-31.6, 48.1)	1007	-11.5 (-43.3, 20.2)	
	**≥0.21**	2422	-17.9 (-41.5, 5.7)	1353	10.9 (-26.4, 48.2)	733	-49.8 (-86.3, -13.4)	
	p for trend[d]		0.135		0.581		0.010	
	p for significance[e]		0.316		0.846		0.023	
**Trimester 1**	***BDCM***							
	**< 0.12**	2670	Reference	743	Reference	1565	Reference	0.122
	**≥0.12 - <0.21**	2325	-18.8 (-41.6, 4.0)	955	4.3 (-35.1, 43.8)	987	-8.6 (-40.6, 23.4)	
	**≥0.21**	2443	-18.5 (-42.2, 5.1)	1346	13.8 (-23.6, 51.2)	746	-44.1 (-80.5, -7.7)	
	p for trend[d]		0.119		0.447		0.025	
	p for significance[e]		0.186		0.739		0.049	
**Trimester 2**	***BDCM***							
	**< 0.12**	2710	Reference	765	Reference	1583	Reference	0.007
	**≥0.12 - <0.21**	2315	-6.8 (-29.8, 16.2)	951	-10.1 (-49.5, 29.3)	971	6.5 (-25.8, 38.8)	
	**≥0.21**	2413	-20.6 (-43.9, 2.7)	1328	7.8 (-29.2, 44.7)	744	-60.8 (-96.5, -25.1)	
	p for trend[d]		0.085		0.584		0.004	
	p for significance[e]		0.215		0.592		0.001	
**Trimester 3**	***BDCM***							
	**< 0.12**	2775	Reference	781	Reference	1597	Reference	0.034
	**≥0.12 - <0.21**	2241	-9.9 (-32.9, 13.0)	912	-4.2 (-43.8, 35.5)	963	-1.2 (-33.2, 30.9)	
	**≥0.21**	2422	-10.2 (-33.4, 13.0)	1351	15.2 (-21.1, 51.6)	738	-48.7 (-84.8, -12.5)	
	p for trend[d]		0.379		0.348		0.017	
	p for significance[e]		0.608		0.500		0.018	

Abbreviations: BDCM, bromodichloromethane; THMBr, total brominated THMs; TTHM, total trihalomethanes. [a]Adjusted for 10 maternal factors (caffeine intake, IMD, education, fasting glucose, post load glucose, ethnicity, smoking, parity, age, BMI) and 2 infant factors (gestational age at delivery as linear and quadratic terms, sex). [b]Ethnic sub-group analyses excluded ethnicity covariate. [c]p-value for significance of exposure-ethnicity interaction term, as a whole, from F-test. [d]p-value for linear trend across tertiles, derived by including the exposure term (coded as 0, 1, 2) as continuous variable in the model. [e]p-value for significance of categorical exposure term, as a whole, within the model, from F-test.

Table S5. Relationship between term birth weight and individual-level joint THM and HAA exposure (DBP7) in Trimester 1, 2 and 3

Exposure	N	TOTAL (n=6529) Adjusted[a] mean difference in term birth weight (g) (95% CI)	N	WHITE BRITISH (N=2651) Adjusted[b] mean difference in term birth weight (g) (95% CI)	N	PAKISTANI ORIGIN (N=2916) Adjusted[b] mean difference in term birth weight (g) (95% CI)	p-value interaction[c]
Trimester 1							
**DBP7 via water consumption (μg/day)[d,e]**							
**<58.6**	2289	Reference	1006	Reference	874	Reference	0.790
**≥58.6 - 97.0**	2112	-11.1 (-35.1, 13.0)	776	-6.6 (-45.2, 32.0)	1073	13.6 (-22.0, 49.1)	
**≥97.0**	2128	11.4 (-13.0, 35.7)	869	29.9 (-7.9, 67.6)	969	35.8 (-1.3, 73.0)	
p for trend[f]		0.386		0.134		0.059	
p for significance[g]		0.202		0.156		0.151	
**Uptake of TTHM via showering, bathing, swimming (μg/day)[d,h]**							
**<0.85**	2269	Reference	649	Reference	1319	Reference	0.489
**≥0.85 - 1.63**	2094	-35.4 (-59.8, -10.9)	827	-25.5 (-68.2, 17.2)	919	-30.9 (-64.9, 3.1)	
**≥1.63**	2166	-26.0 (-51.2, -0.7)	1175	-12.4 (-52.8, 28.0)	678	-47.1 (-85.8, -8.4)	
p for trend[f]		0.049		0.709		0.012	
p for significance[g]		0.014		0.496		0.033	
Trimester 2							
**DBP7 via water consumption (μg/day) [d,e]**							
**<58.6**	2233	Reference	986	Reference	853	Reference	0.081
**≥58.6 - 97.0**	2094	-19.2 (-43.4, 5.1)	785	-30.8 (-69.3, 7.7)	1037	18.0 (-18.4, 54.3)	
**≥97.0**	2202	-4.6 (-28.8, 19.7)	890	19.1 (-18.6, 56.9)	1026	11.8 (-25.1, 48.8)	
p for trend[f]		0.715		0.347		0.566	
p for significance[g]		0.275		0.046		0.618	
**Uptake of TTHM via showering, bathing, swimming (μg/day) [d,h]**							
**<0.85**	2295	Reference	656	Reference	1335	Reference	0.046
**≥0.85 - 1.63**	2077	-7.8 (-32.3, 16.8)	838	-6.2 (-48.8, 36.5)	896	-6.2 (-40.5, 28.2)	
**≥1.63**	2157	-26.5 (-51.4, -1.6)	1157	-1.5 (-41.4, 38.4)	685	-64.3 (-101.8, -26.8)	
p for trend[f]		0.042		0.987		0.002	
p for significance[g]		0.102		0.952		0.002	

Exposure	N	TOTAL (n=6529) Adjusted[a] mean difference in term birth weight (g) (95% CI)	N	WHITE BRITISH (N=2651) Adjusted[b] mean difference in term birth weight (g) (95% CI)	N	PAKISTANI ORIGIN (N=2916) Adjusted[b] mean difference in term birth weight (g) (95% CI)	p-value interaction[c]
Trimester 3							
**DBP7 via water consumption (µg/day)** [d,e]							
**<58.6**	2225	Reference	977	Reference	849	Reference	0.946
**≥58.6 - 97.0**	2127	-10.0 (-34.2, 14.2)	772	8.9 (-29.4, 47.2)	1069	3.3 (-32.9, 39.5)	
**≥97.0**	2177	4.8 (-19.6, 29.3)	902	22.9 (-15.3, 61.1)	998	24.3 (-12.7, 61.2)	
p for trend[f]		0.702		0.243		0.188	
p for significance[g]		0.479		0.490		0.356	
**Uptake of TTHM via showering, bathing, swimming (µg/day)** [d,h]							
**<0.85**	2374	Reference	682	Reference	1357	Reference	0.031
**≥0.85 - 1.63**	2053	-18.8 (-43.0, 5.5)	832	-20.6 (-63.1, 22.0)	894	-21.6 (-55.4, 12.2)	
**≥1.63**	2102	-14.0 (-39.1, 11.1)	1137	9.2 (-30.9, 49.3)	665	-59.5 (-98.2, -20.9)	
p for trend[f]		0.281		0.506		0.003	
p for significance[g]		0.290		0.282		0.009	

Abbreviations: DBP7, sum of TTHM, DCAA, TCAA and BDCAA; HAA, haloacetic acid; THM, trihalomethanes; TTHM, total trihalomethanes. [a]Adjusted for 10 maternal factors (caffeine intake, IMD, education, fasting glucose, post load glucose, ethnicity, smoking, parity, age, BMI) and 2 infant factors (gestational age at delivery as linear and quadratic terms, sex). [b]Ethnic sub-group analyses excluded ethnicity covariate. [c]p-value for significance of exposure-ethnicity interaction term, as a whole, from F-test. [d]Model includes both exposure terms. [e]Consumption via drinking water (µg/day) of sum of TTHM, DCAA, TCAA, and BDCAA. [f]p-value for linear trend across tertiles, derived by including the exposure term (coded as 0, 1, 2) as continuous variable in the model. [g]p-value for significance of categorical exposure term, as a whole, within the model, from F-test. [h]µg/day uptake into blood via these activities.
